# Superheat of silicon crystals observed by live X-ray topography

**Published:** 2004-07-01

**Authors:** Jun-ichi Chikawa

**Affiliations:** Center for Advanced Science and Technology, 3-1-1 Kouto, Kamigori-cho, Ako-gun, Hyogo 678-1205

**Keywords:** Superheat, crystal growth, dislocations, live X-ray topography

## Abstract

*In-situ* observations of Si crystal growth and melting have been carried out by live X-ray diffraction topography. Superheated solid states beyond the melting point was observed for dislocation-free crystals with melting in their inside. Dislocations were found to impede superheat and to melt the crystal without an appreciable superheating. A slightly superheated state accompanying melting removes all dislocations including immobile ones by their climb motion. It is proposed that self-interstitials needed for the volume change by melting are supplied by climb of dislocations, in contrast to dislocation-free crystals creating the interstitials thermally. In real crystal growth, remelting occurs naturally by melt convection and acts to make the growing crystal dislocation-free.

## Introduction

Superheat of solid is known as a state at temperatures beyond its melting point and is not usually observed, i.e., melting can apparently start at crystal surfaces without an appreciable superheating defined by *ΔT* = *T* – *T*_0_ where *T*_0_ is the melting point (*T*_0_ = 1410 °C).

Why does superheat of solids rarely occur, in contrast to the fact that liquid is easily supercooled?

This has been one of the long-asked questions. The liquid and solid states have been believed to be asymmetric with respect to the melting point. Therefore, superheated solid states have not been investigated to the best knowledge of the author. Such non-equilibrium states can be observed with the live X-ray topography that the present author developed.1)

Although a superheated state is imagined to have a crystal lattice with a lot of line imperfections (dislocations) as an intermediate state between crystal and melt, it was found to be a dislocation-free “ideally perfect” state; perfect crystals can be highly superheated, and superheat makes the crystal perfect. Such unanticipated behavior of superheat is important for growing perfect crystals; silicon crystals are grown from the melt near the melting point, and temperature fluctuation due to melt convection causes remelting which is accompanied with superheat of the interface region.

Imperfection of crystals has been one of the main problems in crystallography, since the famous “Laue-Friedrich-knipping” experiment on X-ray diffraction was carried out in 1912. Real crystals have shown intermediary diffracted X-ray intensities between those obtained theoretically for “ideally perfect” and “ideally imperfect” crystals.2) The dislocation theory in the 1950’s came to the conviction that crystals become imperfect easily. Generally, dislocation densities were found to number more than 10^7^ lines/cm^2^ for metals. Therefore, it was widely believed that real crystals contained dislocations in very high densities, as expected from the X-ray diffracted intensities that were much higher than those for an “ideally perfect” crystal. In addition, the famous model “spiral growth” of crystals3) was proposed in 1949 and has led to envisaging that dislocations propagate continuously with forming spiral steps at growth interfaces. However dislocation-free silicon crystals4) were manufactured around 1960 and showed the diffracted X-ray intensity for an “ideally perfect” crystal.5),6) Crystal perfection is very important to realize electronic devices, and efforts to improve crystal quality are still continuing. Today, huge silicon crystals with a diameter of 30 cm and a length of more than 100 cm have been manufactured with a dislocation-free state.

Why do silicon crystals grow into a dislocation-free state?

This question has a close relation with the problem why superheating of solids rarely occur, like that between two sides of the same coin.

## Intrinsic defects

Crystal lattices have point defects as well as line defects (dislocations). Impurity atoms, self-interstitial atoms, and vacancies (vacant lattice points) are considered as point defects. Generally, vacancies are intrinsic defects. Because two types of microdefects are formed by agglomerations of vacancies and self-interstitial atoms in cooling processes of grown silicon crystals, most investigators have believed that both of vacancies and self-interstitial atoms are intrinsic. However, self-interstitials are considered to be extremely unstable; self-interstitials introduced by electron-beam bombardment were found to move even at the low temperature of 4.2 K and to exchange their position with substitutional B, Al, Ga-dopant atoms.7) The surface region of a crystal must have a nearly equilibrium concentration of self-interstitials in almost all of the temperature range in the cooling process, and the formation of interstitial-type microdefects near the side surface of crystals cannot be explained simply as the condensation of self-interstitials and strongly suggests the possibility of some kind of impurity as the origin of the defect.

**Fig. 1:**
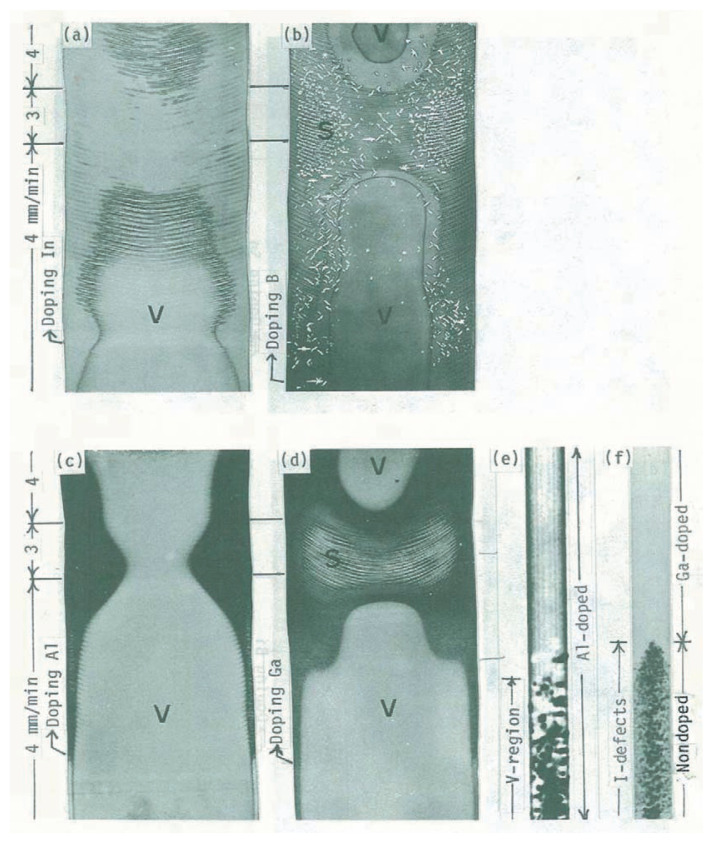
Doping effects of Group-III elements on formation of microdefects. The growth rate was changed as 4→3→4 mm/min. Crystal diameter = 42 mm. The X-ray topographs were taken for longitudinal specimens after Cu-decoration. (a) In-doped, 1.5 × 10^16^ atoms/cm^3^. (b) B-doped, 5 × 10^16^ atoms/cm^3^. (c) Al-doped, 2.2 × 10^17^ atoms/cm^3^. (d) Ga-doped, 2 × 10^17^ atoms/cm^3^. The photographs in (a)–(d) are positive. (e) and (f) are section topographs (negative photographs) for the Al and the Ga-doped crystal, respectively. The interstitial type of defects is not seen by doping Al.

To know which of them is intrinsic,8) effects of trace impurities on microdefects should be clarified because the equilibrium concentrations of intrinsic defects are expected to be very low even at high temperatures near the melting point. For this purpose, Group-III and -V elements, such as B, Al, Ga, In, P, Sb, and Bi, were doped during the float-zone growth of non-doped crystals. The growth rate was changed to generate microdefects, which were so small that copper decoration is necessary for observing by conventional X-ray topography; the specimens were cut longitudinally from the grown crystals and electroplated with copper. After anneal at about 1000 °C for 1 hr, the specimens were quenched to room temperature. By this procedure, copper diffused into the specimen and precipitated at the microdefects, which became visible by X-ray topography. In Fig. 1, topographs for the Cu-decorated specimens doped with In, B, Al and Ga are shown to compare the doping effects. Vacancy-type defects and swirl defects (interstitial-type) are seen in the regions labeled “V” and “S”, respectively. In Fig. 1(a) and (b), V-regions are surrounded by black lines, and the regions outside of the V-regions are seen white. In this white region, the present author discovered extremely small defects in so high densities as to form a white homogeneous background and named “I-defects” with meaning interstitial and impurity.8) The black line is the boundary which is nearly perfect as a result of the annihilation of vacancies and interstitial-type defects.

By doping with aluminum, only a V-region is formed as seen in Fig. 1(c). In the Al- and Ga-doped crystals in Fig. 1(c) and (d), the regions outside the V-regions are black, i.e., they are defect-free. To confirm the crystal perfection, the section topograph9),10) in Fig. 1(e) was taken across the edge of the V-region (Al-doped) in Fig. 1(c); individual defects (vacancy-type) are seen, and the upper part shows straight “Pendellösung fringes” with the “margin effect”,9),10) which indicates a very high quality “ideally perfect” crystal outside the V-region. The section topograph in Fig. 1(f) was taken across the boundary between Ga-doped and non-doped regions near the left edge of Fig. 1(d); interstitial-type microdefects “I-defects” are seen in the lower part, but defects are invisible in the Ga-doped part. No fringe patterns are seen. This suggests a uniform distribution of extremely small defects in a high density.

These observations show the following results. (1) Copper decoration never forms copper precipitates in perfect crystals; precipitates occur only on existing defects. (2) Dislocation-free float-zoned crystals always contain interstitial-type microdefects “I-defects” (3) Al-doped crystals have only vacancy-type microdefects.8) It is emphasized that doping of Al eliminates all interstitial-type defects but does not affect the formation of vacancy-type defects, in spite of the general concept that self-interstitials and vacancies must be related to each other, e.g., their pair formation. This observation indicates that the origin of interstitials is completely different from that of vacancies. The formation of vacancy clusters (cavities) can be understood simply as homogeneous nucleation of supersaturated vacancies during the cooling process. The results indicate that intrinsic defects of Si crystals are only vacancies.

It is well known that doping of Al reduces the impurity oxygen concentration due to formation of Al_2_O_3_ in the silicon melt. Doping Ga also has a similar effect. It is suggested that interstitials originate from trace impurities of oxygen remaining in argon gas atmosphere used for the float-zone method, where oxygen impurities may be resolved and concentrated into the silicon melt.

Quenched crystals in the temperature range from 1380 °C up to the melting point (1410 °C) always showed V-regions.11) Also, Si crystals with a large diameter contain vacancy clusters (cavities) dominantly. It is concluded that the intrinsic defect is vacancy in all the temperature range below the melting point.

## Live X-ray topography

If dynamic observation by X-ray diffraction topography were possible, it would allow one to investigate structural changes occurring over a wide area in real crystals and during changes of environmental parameters such as temperature, stress, and atmosphere. However, X-ray topographic images initially employed only photographic recording with a long exposure time ( 20 min to several hours). Although X-ray sensing television cameras for medical use were available, their resolution was too poor to observe individual dislocations in crystals.

In 1968, we developed an X-ray television system with a high-power rotating-target X-ray generator that could instantaneously display individual dislocations on a TV monitor.12) At the 4th International Conference on Crystal Growth in Tokyo (1974), a movie of X-ray topographic images showing growth processes of Si crystals from the melt was exhibited.13) This type of technique was referred to as “live X-ray topography” 1) and has been used for *in-situ* observations of defects, crystal growth, and phase transformation.

**Fig. 2:**
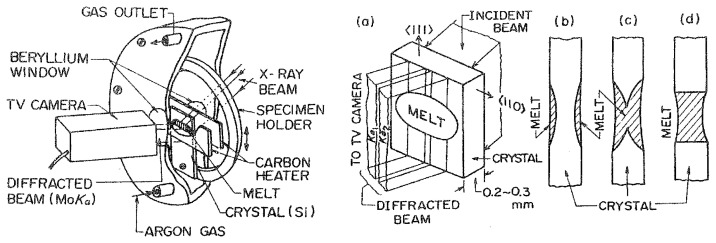
Furnace for the *in-situ* observation of melting and growth processes by live X-ray topography and geometry of a silicon specimen crystal. (a) Orientation of the specimen crystal which is placed so as to satisfy the Bragg diffraction condition for the MoK*α*_1_ and K*α*_2_ radiation. (b)–(d) Cross-section of the specimen in the melting sequences. Crystal growth starts from (d).

**Fig. 3:**
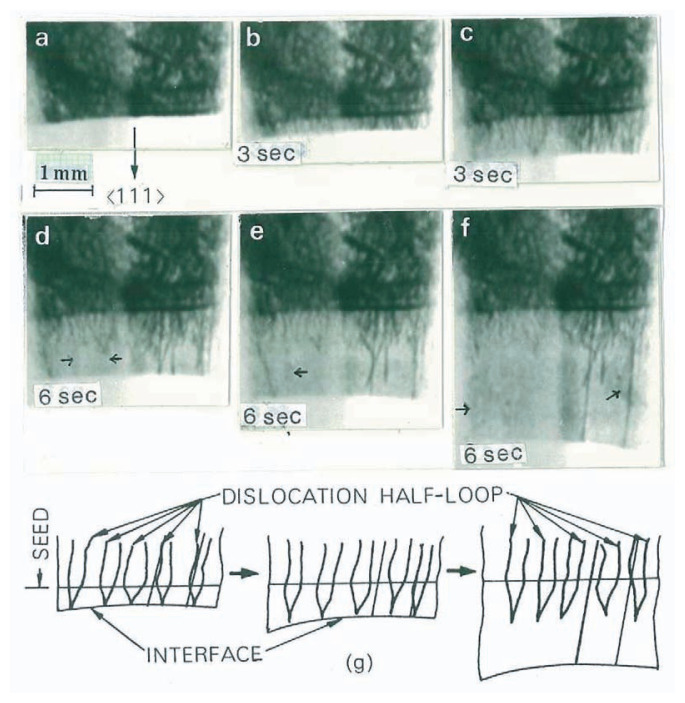
Dislocations in a silicon crystal growing downward from the melt. (a)–(f) Direct-view images with time intervals indicated between the photographs. 220 reflection and a crystal thickness of 0.3 mm. The photographs are negative, and the black lines are dislocation images having higher intensities than the background. Fast moving dislocations have broad images as indicated by the arrows. The right and left halves of each photograph are due to MoK*α*_1_ and K*α*_2_, respectively; their intensity difference was adjusted in printing from the movie film, in order to make the K*α*_2_ image useful. (g) Schematic illustration of the dislocation configurations. The two dislocations perpendicular to the interface are of a composite type consisting of three common dislocations.

Here we show dislocation behavior near the melting point observed by the live X-ray topography. The method is shown schematically in Fig. 2. A plate-shaped Si crystal was placed between two carbon heaters and was oriented to satisfy the Bragg condition for an incident X-ray beam from the Mo-target X-ray generator. The middle part of the crystal was melted and then solidified in an argon gas flow by changing the heat power. Beams diffracted from the crystal were observed through the carbon heater using an X-ray sensitive video camera tube. Crystal imperfections are imaged as “ideally imperfect” parts in an “ideally perfect” background (See Fig. 3).

As seen from Fig. 2(a), two band-shaped regions of the crystal, each of 1-mm width, are imaged instantaneously on the TV monitor by MoK*α*_1_ and K*α*_2_ beams. Such images are called “direct-view images” for convenience. To image a large area, the video signal for K*α*_1_ images are electrically selected and stored in a digital image processor, while the carriage is moved on which the furnace and video camera are mounted. At the end of carriage motion, the imaging area of the camera tube (4 × 6 mm) is displayed on the monitor. Such images are referred to as “synthesized images”.

## Dislocations in growth processes

As a typical example of crystal growth, a sequence of growing downwards is shown with a series of direct-view images from Fig. 3(a) to (f). Because there was no diffracted intensity from the molten part, solid-liquid interfaces were seen clearly, even at the melting point *T*_0_ = 1410 °C, as the lower edge of each image. Figure 3(a) shows the state of the crystal just before the growth starts. The crystal image is black due to the many dislocations generated during the temperature increase. Dislocations exist stably at the interface in equilibrium, where the temperature *T* at the interface is equal to the melting point, i.e., *T* = *T*_0_ and growth rate *V* = 0. In the growth process shown in Fig. 3(b)–(f), the dislocations follow the moving interface, forming hairpin-shaped half-loops behind the interface (*T* < *T*_0_). With increasing growth rate, the majority of dislocations are left behind the interface, and dislocation-free regions are formed. These dislocations have a common type of Burgers vector, 1/2<110>. This observation indicates that dislocations are unstable at growth interfaces with a supercooling defined by Δ*T* = *T*_0_ – *T* > 0, except the two dislocations perpendicular to the interface seen in Fig. 3(f). They are identified to be immobile composite dislocations consisting of three common dislocations.

Why are common dislocations unstable at growth interfaces?

In general, a dislocation must intersect with the interface to minimize its total length (free energy) at the equilibrium at *T* = *T*_0_. Interfaces are always covered with the most stable facets {111} even if it looks smoothly curved. When the interface is supercooled, an increase in free energy of the interface is caused by the presence of the dislocation that newly forms a spiral step on the microscopically facetted surface. Consequently, the dislocation is forced away so as to minimize the interfacial free energy. The total length of the spiral step is proportional to the supercooling, Δ*T* = *T*_0_ – *T*.14) If the increase in free energy due to formation of the spiral step exceeds that due to formation of a dislocation loop near the interface as shown schematically in Fig. 3(g), the dislocation cannot exist at the interface.

When dislocation density increases, steps formed by neighboring dislocations with opposite signs annihilate each other, and therefore they can exist at the interface. However, a simple calculation shows that the dislocation density required is fairly high and one can conclude that growth interfaces at *T* < *T*_0_ always destabilize the common dislocations that could form spiral steps on the interfaces, unless they form immobile composite types.14)

To grow a dislocation-free crystal, the problem is how to eliminate composite-type dislocations. Especially, an edge-type dislocation with a Burgers vector perpendicular to the growth direction remains stable at the growth interface, because of no forming of new steps. This is the case of the two composite dislocations in Fig. 3(f).

In industrial crystal growth, silicon (melting point *T*_0_ = 1410 °C) is molten in a quartz crucible, and then a small cylindrical seed crystal is attached to the melt surface. After that, the seed was pulled up with solidifying the melt into a large-diameter crystal. In the light of the observations in Fig. 3, one can understand the configurations of dislocations observed for the seed part of dislocation-free crystals. Irregularly curved common dislocations are located only in the seeding zone. Although they do not propagate, composite dislocations are formed depending upon the density of common dislocations. Thus, the initial narrow crystal grown from the seed has only composite-type straight dislocations14) consisting of three common dislocation such as [111] = 1/2[110] + 1/2[101] + 1/2[011] and [100] = 1/2[110] + 1/2[101] + 1/2[011]. They were formed by elastic interaction among three dislocations moving on different slip planes and became immobile.

Is there any method to eliminate the immobile dis-locations?

## Dislocation effect on melting

Melting in a dislocation-free state had been considered to be extremely difficult compared with dislocation-free crystal growth. Dislocations were always generated in silicon crystals during a temperature increase up to the melting point. The *in-situ* topographic observation showed that dislocation sources are located at the edges of specimen crystals. By sharpening the crystal edge to a knife-edge, dislocation-free melting was realized.

**Fig. 4:**
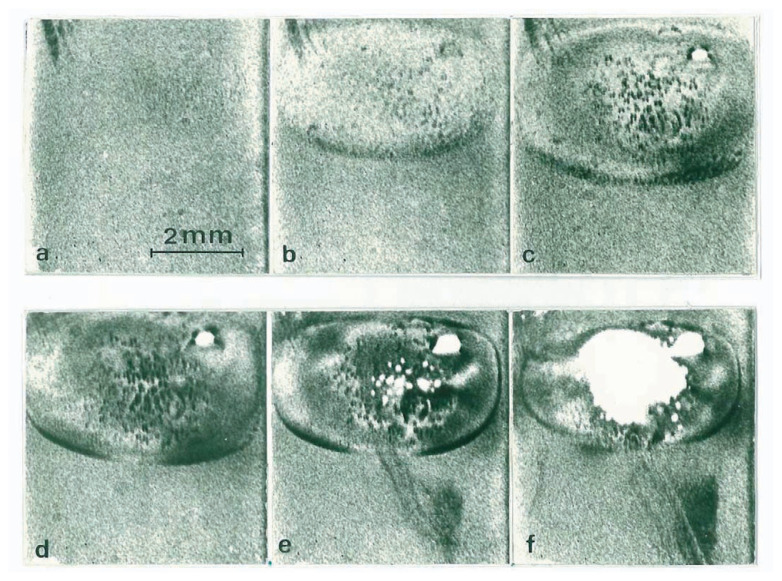
Synthesized images showing a melting sequence of a dislocation-free crystal. 220 reflection. 10-sec intervals. The elliptical region is melting from the surfaces and some Pendellösung fringes are seen due to the thickness variation. Black spots in the central region of the ellipse are due to liquid drops inside the crystal. Although dislocations are generated in the ellipse of (e), all of them move downwards, and the ellipse becomes dislocation-free.

The melting process of a dislocation-free crystal is shown by the series of direct-view images in Fig. 4. Figure 4(a) shows the state just before melting. In Fig. 4(b), melting from the surfaces is initiated in the elliptical region. The temperature is highest at the center of the ellipse where black spots are seen. In Fig. 4(c), melting has proceeded farther, and many black spots are seen. The uneven intensity distribution inside the region is that of equal-thickness fringes (Pendellösung fringes)10) due to the variations in crystal thickness shown in Fig. 2(b) and (c). It is surprising that melting crystals are “ideally perfect” so as to cause multiple X-ray scattering. In Fig. 4(d)–(f), on decreasing the heating power slightly, the ellipse shrinks, i.e., growth takes place at the periphery of the ellipse, but melting proceeds at its central region due to thermal inertia; the crystal becomes thinner at its center in Fig. 4(d)–(e). Some black spots become white in Fig. 4(e) and are holes formed by melting through the crystal. (The photographs are negative, and the diffracted intensity is zero in the white regions.) The black spots are due to strains around liquid drops formed inside the crystal. Melting through, as shown schematically in Fig. 2(c), takes place in Fig. 4(f).

It should be noted in Fig. 4(e) that some dislocations are generated from drops but they are unstable and immediately move away from the elliptical melting region. The direction of the dislocation motion is quite different from those of {111} slip planes; the dislocations move downward by climb motion. The motion was so fast as to occur at an instant during the 10-sec interval between Fig. 4(d) and (e).

The observation of drops inside crystals means that dislocation-free crystals can be superheated, because drop formation requires a fairly large superheating to overcome the solid-liquid interfacial free energy, even if the drops are formed by some nucleation centers.

**Fig. 5:**
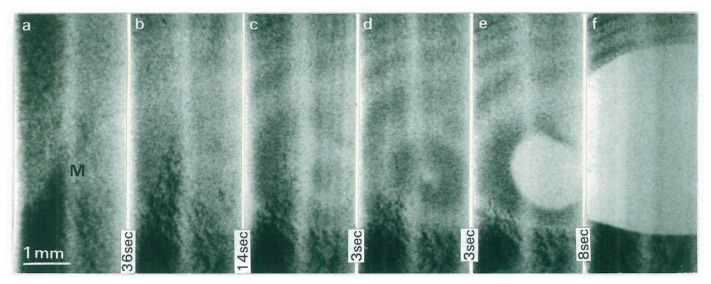
Direct-view images showing a melting sequence of a dislocated crystal. 220 reflection. Each images consists of two bands due to the MoK*α*_1_ and K*α*_2_. Time intervals are indicated between the photographs. The marked “M” has the highest temperature, and the temperature gradient is high in the lower part.

Then, it is questioned whether or not dislocated crystals are superheated. The melting behavior of a dislocated crystal is seen in the series of photographs in Fig. 5. Figure 5(a) shows the state just before melting starts; the black parts are due to many dislocations generated on heating the crystal to the melting point. The point marked “M” has the highest temperature, and the darkness of the region “M” in Fig. 5(a) becomes lighter in Fig. 5(b), i.e., dislocation densities are decreased rapidly before melting. In Fig. 5(c)–(e), the circular equal-thickness fringes expand from Point M with thinning of the crystal by melting homogeneously from the surface.

This observation shows that the dislocations move away from the melting region toward the lower-temperature regions, as have been seen in Fig. 4(e) and the crystal becomes “ideally perfect” just before melting; a large volume of the crystal is dislocation-free. The observation in Fig. 5 shows that a dislocated crystal melts homogeneously from its surface with forming a dislocation-free state with no drops. This result means that dislocated crystals melt without an appreciable superheating, i.e., dislocations impede superheat of the crystal.

## Effect of superheating on dislocations

**Fig. 6:**
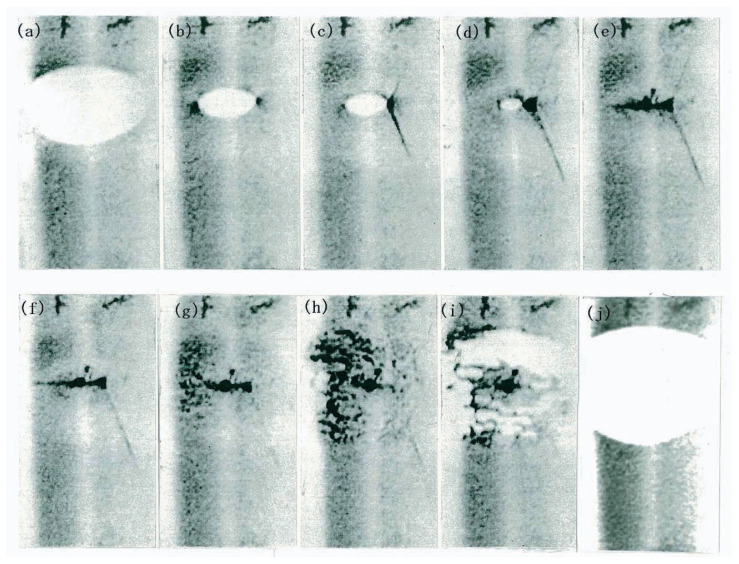
Direct-view images showing a successive process of growth and melting by the upper (a)–(e) and the lower series (f)–(j), respectively. 220 reflection. The time length from (a) to (j) is about 70 sec. The intervals between images are arbitrary. The width of each photograph corresponds to 3 mm on the crystal. The photographs are negative. (a) Growth with shrinking of the white elliptical molten region. (b) Stress concentrations by the smaller molten region. (c) Two slip bands generated. (d) Propagation of the slip bands upwards and downwards. (e) Termination of the growth with generation of dislocations. (f) Start of melting again by increasing the heating power. (g) The slip bands fade out in the elliptical superheated region with liquid drops on the upper-left. (h) Generation of liquid drops in the superheated region. The slip band remains outside the superheated region. (i)–(j) Melting through the crystal.

Although the temperature difference between growth and melting is only a few degrees in centigrade, dislocations are much more unstable in melting processes than in growth processes. To demonstrate the difference between the states lower and above the melting point, growth and melting of a crystal were repeated several times and observed by the live X-ray topography, as shown in Fig. 6. An elliptical region of a crystal was melted, and, by decreasing the heating power slightly, dislocation-free growth takes place so as to shrink the molten elliptical region, as seen from the series of direct-view images in Fig. 6(a) to (e). Two slip bands are generated at the corner of the shrinking ellipse owing to stress concentration in Fig. 6(c) and propagate on the {111} slip planes upwards and downwards, as seen from Fig. 6(c)–(e). The growth terminates with generation of some localized dislocations by solidifying the entire of the molten region in Fig. 6(e). By increasing the temperature to melt again, a remelting process is seen from the series of images in Fig. 6(f)–(j). The equithermal lines must be elliptical, similarly to those in the growth process. The images of the slip bands generated in the growth process are being faint; the upper slip band disappears in Fig. 6(f)–(g), and the lower one disappears inside the elliptical superheated region, as seen from Fig. 6(g). Simultaneously liquid drops are formed in the dislocation-free region on the left side of the ellipse [Fig. 6(h)]. Although the strong strain fields around drops seen in Fig. 6(h) can be considered as active sources of dislocation generation, it is remarkable that no dislocations are generated in a superheated state.

In Fig. 6(f)–(g), the fading of slip bands takes place uniformly in the superheated region, and the lower slip band remains outside the region. This observation indicates that dislocations in the slip bands move away from the crystal surfaces. Since each dislocation in a slip band exists through the crystal from surface to surface, their disappearance from the surfaces must be by their climb motion. In Fig. 6(i), melting occurs unevenly as a typical case of a dislocation-free state, and an elliptical molten region is formed in Fig. 6(j). The interface is dislocation-free. Therefore, by changing the heating power, the growth and melting can be repeated in the same behavior as Fig. 6(a)–(e). This means that all dislocations generated in growth are removed from the interface by each remelting.

**Fig. 7:**
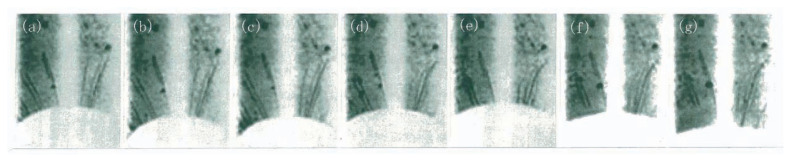
Removing composite dislocations by remelting introduced with a pulsed heating during the growth. The total sequence of direct-view images is about 2 min. The width of each photograph corresponds to 3 mm on the crystal. The crystal is growing downward in (a)–(c), remelting in (c)–(e), and growing again in (e)–(g). Several composite dislocations existing at the interface stop their propagation by remelting, and dislocation-free interface is seen in (f) and (g), except a hairpin-shaped dislocation loop on the right. The loop is formed from two composite dislocations with the opposite signs connecting near the melting interface by their climb motion in (e) and looks to reach the growth interface in (f).

Composite dislocations are anchored against slip motion and can exist stably at growth interfaces. It should be examined whether they can be removed by superheating. The effect of remelting during growth was observed as shown in Fig. 7. In a growth process from Fig. 7(a) to (c), there are seen several composite dislocations reaching the round interface and propagate stably into the newly grown part. By increasing the heating power, remelting is seen from Fig. 7(c) to (e), and by decreasing the temperature, regrowth occurs from Fig. 7(e) to (g). It is seen that their propagation is stopped except a hair-pin shaped loop, and a dislocation-free region along the interface is formed in Fig. 7(f) and (g). This observation shows that composite dislocations move out from the surfaces by climb motion. One can conclude that remelting causes climb motion even for composite dislocations and remove them. No drops are seen in the remelting process. This indicates that a small superheating still has the effect.

## Superheated states

The experimental results described above are summarized as follows:

1. The intrinsic defect in silicon crystals is vacancy at the melting point and under, *T*≦*T*_0_.
2. At equilibrium between the melt and crystal, *T* = *T*_0_, dislocations exist stably at the interface.
3. In growth processes, *T* < *T*_0_, common dislocations cannot reach growth interfaces if their Burgers vectors have a component parallel to the growth direction. Composite dislocations (immobile) consisting of three common dislocations can stay at growth interfaces. Especially, the edge-type dislocations exist stably perpendicular to the interface. Therefore, to grow a dislocation-free crystal, their removal from the interface is necessary.
4. Dislocation-free crystals can be superheated with liquid drops inside them; *T* > *T*_0_.
5. Dislocated crystals melt homogeneously from the surface with making the interface region dislocation-free. No drops are formed because of a smaller superheating; *T* ~ *T*_0_. Dislocations impede superheating.
6. In superheated states *T* > *T*_0_, no dislocations are generated even if there are generation sources having strongly distorted regions such as drops and dislocations piled up.
7. Composite dislocations can be removed by superheating slightly with remelting.
8. Dislocations move by slip motion at *T*≦*T*_0_ and by climb motion at *T* > *T*_0_.

These observations are arranged in Table I and lead to the following argument.

Owing to the volume change accompanied by the solid-liquid phase transition (the liquid is denser by 9%), a superheated crystal needs self-interstitials to proceed toward melting (the intrinsic defect is vacancy at *T*≦*T*_0_). Dislocations can supply easily the interstitials by consuming their extra-half planes from the dislocation cores, i.e., by climb motion. (The pure screw-type of dislocations become helical by climb motion). Thus, all dislocations are eliminated from superheated regions, and sometimes pile up at the edge of the regions.15) This also means that dislocations impede superheating.

In contrast to dislocated crystals, dislocation-free crystals have so high superheating as to create interstitials thermally in the perfect state, and melting in their inside occurs by forming drops as agglomerates of the interstitials.

In real crystal growth, remelting takes place easily due to the melt convection in the seeding process. As has been seen in Figs. 6 and 7, superheated states accompanied by remelting can remove all dislocations including immobile dislocations. To answer the question why silicon crystals grow into a dislocation-free state, it is proposed that the dislocation-free state of silicon crystals is formed due mainly to superheat with remelting which naturally takes place during growth.

In the steady-state growth of the main crystal part with a large diameter, remelting should be avoided because remelting often causes formation of microdefects such as swirls.

The knowledge on superheat is useful for silicon industry. Today, dislocation-free huge crystals with a diameter of 30 cm and a length of more than 1 m are manufactured as a main product. The seed crystal must be thick enough for pulling up such a heavy crystals, and the seeding technique may be realized by introducing remelt artificially.

## Conclusion

The conclusion is summarized as follows: (1) Dislocation-free crystals can be so superheated that melting takes place in their inside. (2) All dislocations are removed with their climb motion by superheating slightly. (3) Dislocations impede superheating.

Now it is questioned whether the defect behavior in superheated states revealed for silicon crystals is expected for metals and other materials. For a metal, its density reduces by melting, in contrast to the case of silicon, and their intrinsic defects are vacancy. Superheat of metals may take place, if they are dislocation-free, by necessity of creating high vacancy concentrations far beyond the equilibrium one for melting. However, metals usually have dislocations in so high densities that suppress superheating.

The long-asked question can be answered by the fact that crystals in nature contain dislocations at high densities.

## Figures and Tables

**Table I:** Defect behavior observed above and below the melting point

Interface	Intrinsic defect	Dislocation		

		stability	motion	generation
melting *T* > *T*_0_		No exist.	Climb	No
equili. *T* = *T*_0_	Vacancy	stable		
growth *T* < *T*_0_	Vacancy	unstable	Slip	Slip bands
